# Self‐Chosen Music as a Contributor to Music‐Induced Analgesia Across Diverse Socio‐Cultural Backgrounds: A Crossover Randomised Controlled Trial

**DOI:** 10.1002/ejp.70095

**Published:** 2025-08-07

**Authors:** Antonia S. Becker, Emilie S. van der Valk Bouman, Julian Schaap, Koen van Eijck, Zahra Bierman, Charlotte Saat, Hans Jeekel, Cecile C. de Vos, Markus Klimek

**Affiliations:** ^1^ Department of Neuroscience Erasmus Medical Center Rotterdam the Netherlands; ^2^ Department of Arts and Culture Studies Erasmus University Rotterdam the Netherlands; ^3^ Centre for Pain Medicine Erasmus Medical Centre Rotterdam the Netherlands; ^4^ Department of Anesthesiology Erasmus Medical Center Rotterdam Rotterdam the Netherlands

**Keywords:** heart rate variability, music, music‐induced analgesia, pain, socio‐cultural background

## Abstract

**Background:**

Music‐induced analgesia (MIA) has been extensively studied, but the role of socio‐cultural background, which shapes music preferences and perceptions, remains unexplored. This study investigates whether the effect of preferred music on pain endurance remains consistent across different socio‐cultural backgrounds.

**Methods:**

In a crossover randomised controlled trial (*n* = 84), participants underwent three auditory interventions in a randomised sequence: self‐chosen music, researcher‐chosen (classical) music and a podcast (control). After 20 min, pain endurance was tested by increasing electric stimuli while participants continued listening. Additionally, pain threshold, perceived pain scores, heart rate variability (HRV) and emotions were measured.

**Results:**

Compared with the control condition, self‐chosen music significantly increased pain endurance (*β* = 0.74, *p* = 0.003) and reduced pain intensity (*β* = −0.43, *p* < 0.001) and unpleasantness (*β* = −0.38, *p* = 0.007). Both self‐chosen (*β* = 0.32, *p* = 0.034) and researcher‐chosen music (*β* = 0.37, *p* = 0.018) increased the pain threshold compared to the control condition. These pain measurements were not influenced by participants' socio‐cultural background (cultural capital, level of education). HRV analysis revealed more parasympathetic activity with the researcher‐chosen music and higher heart rate with the self‐chosen music than with the control intervention.

**Conclusions:**

Listening to self‐chosen music was effective in MIA, irrespective of socio‐cultural background or type of music. In contrast, researcher‐chosen (classical) music was most effective in enhancing parasympathetic activity. This study highlights that self‐selected music in line with personal preferences is effective for pain relief, encouraging personalised approaches in MIA.

**Significance Statement:**

This study revealed that self‐chosen music significantly enhances pain endurance and reduces subjective pain scores, regardless of socio‐cultural background and type of music. In contrast, researcher‐chosen (classical) music increases parasympathetic activity, indicating a more ‘relaxed’ state. These findings suggest that different types of music may serve different functions, with self‐chosen music being especially effective for pain relief. Encouraging patients to self‐select their music could improve pain management outcomes, underscoring the importance of personalised approaches when using music for pain relief.

## 1 Introduction

The ability of music to influence pain perception, also known as music‐induced analgesia (MIA), has been increasingly investigated in recent years. In medicine, randomised controlled trials and meta‐analyses have demonstrated that music interventions can reduce pain, anxiety, stress responses and opioid requirements across various healthcare settings (Fu et al. 2020; Hole et al. 2015; Kakar et al. 2021; Kühlmann et al. 2018; Lee 2016; Song et al. 2024; Umbrello et al. 2019). However, both experimental studies and clinical trials show high heterogeneity regarding music selection, music characteristics and participants' backgrounds. At present, there is no consensus on the most effective type of music for specific purposes and patient groups.

Previous experimental studies indicate that self‐selecting music in line with individual preferences is most effective in MIA (Basinski et al. 2021; Howlin and Rooney 2021a; Valevicius et al. 2023; Van der Valk Bouman et al. 2024). Nonetheless, clinical studies often utilise music selected by the researcher, with a predominant focus on Western classical music (Hole et al. 2015; Kühlmann et al. 2018). Although the neurobiological underpinnings of MIA are not yet fully understood, factors such as changes in emotions and cognitive set, individual beliefs and the autonomic nervous system might underlie the effectiveness of (self‐selected) music (Koelsch and Jäncke 2015; Lunde et al. 2019; Vuust et al. 2022). Direct comparisons between different types of music, such as self‐chosen and researcher‐chosen, with suitable control conditions and consideration of underlying mechanisms, remain scarce.

Moreover, most experimental studies on MIA have been conducted in Western countries with predominantly highly educated participants, raising questions about the generalisability of previous findings to more diverse populations (Lee 2016). Medical research indicates a crucial role of socioeconomic status and cultural background in pain perception and management (Fillingim 2017; Meints et al. 2019, 2016). Additionally, previous research has established that personal characteristics such as gender, ethnicity, status and individual values contribute to differences in music preferences and perceptions, which could in turn affect MIA (Bourdieu 1984; Cowen et al. 2020; Ter Bogt et al. 2011). For instance, cultural capital acts as a key factor in music taste formation, consequently shaping individual listening experiences (Jæger and Møllegaard 2022; Van Eijck 2001). Cultural capital refers to the nonfinancial cultural resources an individual possesses, which have typically been inculcated since childhood and are therefore strongly affected by their parents' socio‐cultural background (Jaeger and Breen 2016; Van der Waal et al. 2024). Typically, higher levels of cultural capital tend to imply greater appreciation for ‘highbrow’ genres such as classical and jazz music (Lizardo and Skiles 2016). Moreover, the context or situation can influence the type of music that people choose (Sonnett 2020). Therefore, investigating MIA among people from different socio‐cultural backgrounds may enable us to utilise music in healthcare more effectively, reflecting the diversity of the actual patient population.

To date, no research has assessed the influence of socio‐cultural background and cultural capital on MIA. To address this gap, we designed a crossover randomised controlled trial to investigate whether the effect of (self‐selected) music on pain endurance remains consistent across different socio‐cultural backgrounds. To explore this, we propose two hypotheses. First, we hypothesise that the positive impact of self‐chosen, preferred music on MIA is consistent irrespective of socio‐cultural background. Second, and in contrast, we hypothesise that researcher‐chosen (classical) music has a universal beneficial impact on MIA.

## 2 Methods

### 2.1 Study Design

**FIGURE 1:**
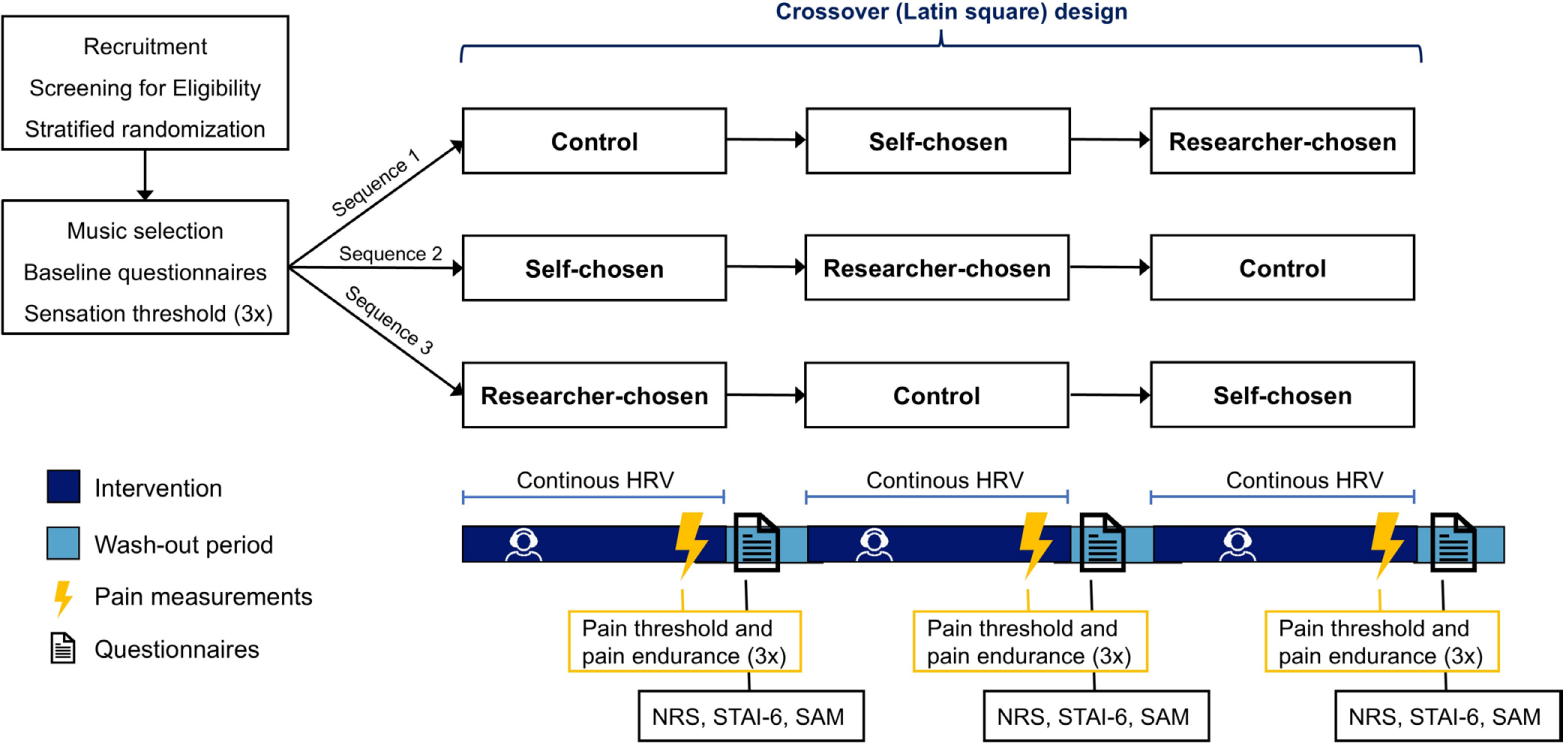
Study design. The figure illustrates the Latin‐square crossover design of the study. After recruitment and randomisation, participants completed comprehensive baseline questionnaires (including questions about (parental) cultural capital, medical history and everyday music listening behaviour). Additionally, the sensation threshold was measured. Subsequently, each participant listened to the three interventions (self‐chosen music, researcher‐chosen music and control) in a randomised order (sequence 1, 2 or 3) for 20 min with headphones. At the end of each intervention, pain threshold (mA) and pain endurance (mA) were measured. Between the interventions, a 20‐min washout period took place, in which pain intensity (NRS), pain unpleasantness (NRS), STAI‐6 and SAM questionnaires were conducted. HRV was measured continuously during the three interventions. At the end of the experiment, personal experience of listening to music and the podcast was evaluated with open‐ended questions. HRV, heart rate variability; mA, miliampere; NRS, numeric rating scale; SAM, Self‐Assessment Manikin; STAI, Spielberger State–Trait Anxiety Inventory.

The MOSART study is a crossover randomised controlled trial conducted from August 2023 to November 2024 at the outpatient clinic of the Center of Pain Medicine at Erasmus Medical Center, Rotterdam, The Netherlands. The protocol was approved by the Medical Ethics Review Board of Erasmus MC (MEC‐2023‐0253) and was preregistered at www.clinicaltrials.gov (NCT06008951). The published protocol provides additional information on the study design and rationale (Becker et al. 2024). Each participant received the same three interventions (self‐chosen music, researcher‐chosen music and control) in a randomised order (Latin square design). Listening to an informative podcast served as a control condition. Each auditory intervention lasted 20 min, after which pain endurance was tested while participants continued listening. In between, a wash‐out period of 20 min was completed. At the end of each intervention, pain endurance by increasing electric stimuli was tested as the primary outcome of the study. Furthermore, as secondary outcomes, pain threshold, perceived pain scores (pain intensity and unpleasantness) and heart rate variability (HRV) were measured and psychological questionnaires—evaluating emotions and anxiety—were conducted. Moreover, a comprehensive survey regarding socio‐cultural background was completed. Due to the nature of the study and the interventions, participants and investigators were not blinded. Figure 1 provides a comprehensive overview of the study design.

### 2.2 Study Population

A sample size of *n* = 84 was calculated based on a previous meta‐analysis, but under consideration of a slightly lower effect size (Cohen's *d* = 0.4) because a podcast was used as a nonmusic control condition (Kühlmann et al. 2018). The study used stratified recruitment and randomisation based on education level to ensure a diverse sample in terms of socio‐cultural background, as education is a key indicator of cultural capital (Coulangeon 2020). Randomisation and stratification were performed using Castor EDC (Ciwit BV., Amsterdam). The sample included three equal parts of less‐educated, medium‐educated and more‐educated female subjects. These groups were based on the International Standard Classification of Education (UNESCO Institute for Statistics 2012).

Heterogeneity in pain endurance is for a large part explained by sex. For example, studies have shown that females are more sensitive to electric stimuli and that their pain threshold is more affected by music (Fillingim et al. 2009). Therefore, only female subjects were included in this study and were exclusively treated by female researchers, to prevent sex/gender‐related differences in pain measurements. Next, only adults up to the age of 60 were included, since significantly reduced responsiveness in autonomic activity is observed in elderly individuals, potentially influencing HRV measurements. Thus, this study included healthy female subjects aged 18–60 years. A full overview of the inclusion and exclusion criteria can be found in Table S1. Participants provided written informed consent and received financial compensation for their time investment.

### 2.3 Interventions

This study used three types of auditory interventions: self‐chosen music, researcher‐chosen music and control. The self‐chosen music was selected by participants in advance, with the instruction to select approximately 10 tracks with the aim of reducing pain. The researcher‐chosen playlist was selected by our research group and consisted of popular classical music (Table S2). The control condition was a neutral podcast that focused on informative topics about flora and fauna and was selected to be nonmusical and devoid of political or emotional content. The music was played via Spotify and noise‐cancelling headphones (JBL Tune 770 NC). The researcher‐chosen playlist was always played in the same order, whereas the self‐chosen music was played in shuffle mode.

### 2.4 Measurements

#### 2.4.1 Pain Measurements

The primary outcome of this study was pain endurance, quantified in (mA), through increasing electric stimuli administered at the end of each auditory intervention (self‐chosen music, researcher‐chosen music and control). The electric stimuli were administered by the STMISOLA (Biopac Systems Inc. Goleta, CA, USA). Two electrodes were attached to the index finger of the nondominant hand. Administration of the electrical pulses of 0.2 ms at a frequency of 100 Hz began when the participants pressed a button with their dominant hand. These electric pulses were administered with a starting intensity of 0 mA, increasing in intensity at a rate of 0.5 mA/s, up to a maximum of 30 mA for safety reasons. Prior to the experiment, the sensation threshold was tested. At the end of each intervention, while participants were still listening to the auditory intervention, the pain endurance was tested, with participants being instructed to hold down the button for as long as possible until they could no longer endure the pain. As secondary outcomes, pain threshold (measured in mA) and perceived pain scores (pain intensity and unpleasantness) were assessed at the end of each intervention. Pain threshold was defined as the point at which participants indicated that the stimulus became painful (whereas pain endurance was defined as the moment at which the participants stopped holding the button for the pain stimulus). Each stimulus was repeated three times and averaged. After each stimulus, participants rated the pain intensity and unpleasantness using an 11‐point numeric rating scale (NRS).

#### 2.4.2 Physiological and Psychological Assessment

Other secondary outcomes of this study were HRV, anxiety levels measured by the State–Trait Anxiety Inventory (STAI‐6), emotions measured by the Self‐Assessment Manikin (SAM) and a music listening questionnaire at the end of all interventions. First, throughout each listening intervention and pain stimuli procedure, HRV was continuously monitored. HRV, which measures the time variation between consecutive heartbeats, is an indicator of autonomic function. HRV was tracked using an Acentas Chest Strap (BM innovations GmbH), which provides an objective assessment of the physiological responses to the interventions. Emotional changes were also assessed because of their role in pain perception. Anxiety levels were measured with the STAI‐6 at baseline and after each intervention. Emotions were further evaluated using the SAM at the same time points, assessing emotional valence and arousal. Additionally, participants' experiences with the music and podcast were evaluated through five open‐ended questions at the end of the experiment. The open answers for the podcast were coded as ‘like’ or ‘dislike’.

#### 2.4.3 Socio‐Cultural Background and Music Characteristics

Before the experiment, participants' demographic characteristics (e.g., age, medical history and handedness) and socio‐cultural backgrounds were assessed using validated questionnaires. These sociological questionnaires focused on personal and parental cultural capital (Van der Waal et al. 2024). Specifically, participants completed a questionnaire evaluating their level of education, occupation, income, ethnicity and both their personal and parental cultural capital. Parental socioeconomic status was assessed, as this serves as a key determinant of early socialisation. Additionally, music listening behaviour was also evaluated, which included weekly hours of passive and active listening, the importance of music and whether participants played instruments or were professional musicians. All questions were based on recent literature and are described in more detail in the protocol paper of this study (Becker et al. 2024; Van der Waal et al. 2024).

The self‐chosen and researcher‐chosen music was analysed via the Spotify application programming interface (API). The definitions of the audio features as described by Spotify are provided in Table S3. The subgenres provided were reduced to 15 genres (Blues, Country, Classical, Easy listening, Electronic, Folk/Acoustic, Hip‐Hop, Jazz, Latin, Metal, New Age, Pop, Rock, R&B, Word/Traditional) via a genre‐reducing algorithm as described in previous literature (Chosic. List of Music Genres; Scarratt et al. 2023). Descriptive statistics were calculated using weights to account for the number of tracks per participant.

### 2.5 Statistical Analysis

Data were validated using Castor EDC (Ciwit BV., Amsterdam) and analysed with R‐Studio (version 2023.12.0) and R (version 4.3.2), utilising the following packages: dplyr, tidyr, ggplot2 and nlme. HRV parameters were calculated using Kubios HRV Scientific (version 4.1.2) (Tarvainen et al. 2014). The time domain included the heart rate (HR) mean, root mean square of successive differences (RMSSD) and standard deviation of NN intervals (SDNN). For the frequency domain, the low‐frequency (LF), high‐frequency (HF) and LF/HF ratios were calculated. Because of the nonconstant HR, RMSSD and SDNN were corrected using the coefficient of variation (cvRMSSD and cvSDNN) by accounting for the mean RR interval of the corresponding time period (de Geus et al. 2019). A description of the HRV parameters is provided in Table S4.

The distributions of all variables were evaluated using data visualisation (histograms and Q–Q plots) and Shapiro–Wilk tests. For normally distributed numeric variables, the mean and standard deviation (SD) were used. Percentages were used for categorical variables. The primary outcome of this study, pain endurance, was analysed using a linear mixed model (LMM), considering sequence and type of intervention. This method accounted for the crossover design of the study. Random slopes were included based on information criteria and log‐likelihood tests. Similarly, secondary outcomes (pain threshold, pain intensity, pain unpleasantness, SAM, STAI‐6 and HRV) were analysed using LMMs. Post hoc pairwise comparisons were conducted for the pain measurements and psychological measurements using the estimated marginal means derived from the fixed effects of the LMMs.

An exploratory approach was employed to investigate the influence of socio‐cultural background on MIA across the different interventions. Socio‐cultural background parameters were based on established literature and included personal cultural capital, parental cultural capital and level of education (Jaeger and Breen 2016; Van der Waal et al. 2024). Personal cultural capital was measured using the sum of the following three items: ‘attending a classical concert, opera, or ballet performance’, ‘visiting a (visual) art museum’ and ‘attending a theatre performance (excluding musicals)’, based on a recent large‐scale sociological study that utilised and validated these three items to measure cultural capital (Van der Waal et al. 2024). Participants rated the frequency of these activities on a 7‐point Likert scale ranging from ‘never’ to ‘more than once a week’. Parental cultural capital was measured similarly, using the same three items to assess how often respondents' parent(s) or caregiver(s) engaged in these activities during the respondent's childhood, specifically between the ages of 12 and 14. Level of education was based on the Dutch education system (UNESCO Institute for Statistics 2012). The socio‐cultural background parameters were subsequently included as additional interaction terms in LMMs to evaluate their influence on pain endurance and the other pain measurements across intervention groups. Finally, simple linear regressions were used to model the effects of cultural capital and level of education on pain endurance.

## 3 Results

**FIGURE 2:**
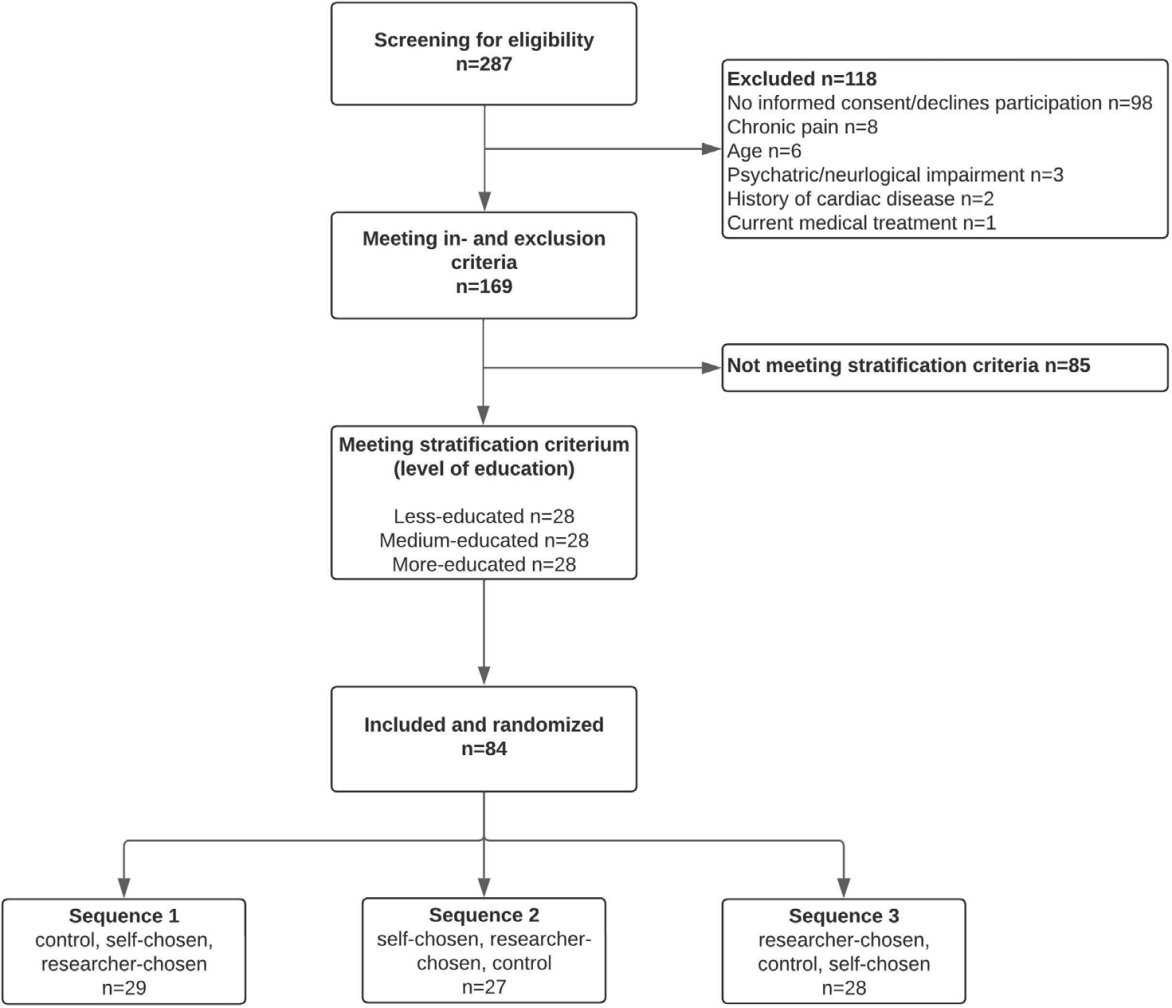
CONSORT flow diagram. The three sequences represent the order in which participants listened to the three interventions.

In total, 84 female participants were included in this study (Figure 2). The baseline characteristics of the study population are shown in Table 1. The average age was 38 years (SD = 13.5, range 18 to 60). In line with the stratification criteria, participants were diverse in terms of education level. Approximately half (55%) of the participants were employed and almost one‐third (32%) had a migration background. Participants reported overall lower to average monthly personal incomes, predominantly in the categories ‘EUR 1500 and less’ (36%) and ‘EUR 1501 to 2500’ (27%), which are considered low to average for the Netherlands. Parental income between the ages of 12 and 14 years was relatively evenly distributed, with most participants indicating average (33%) parental incomes. In general, participants expressed a high music importance (6.2 on a 7‐point Likert scale).

**TABLE 1:** Baseline characteristics of the study population.

Category	Value (*n* = 84)
Age (years) *mean (SD)*	38.2 (13.5)
Level of education^a^ *n* (%)	
Primary and junior vocational education	5 (6)
Junior general secondary education	23 (27)
Senior general secondary and intermediate vocational education	24 (29)
Preuniversity education	4 (5)
Vocational colleges	9 (11)
University	19 (23)
Current occupation *n* (%)	
Student	16 (19)
Self‐employed	9 (11)
Employed	46 (55)
Job seeking	3 (4)
(Partially) disabled	6 (7)
Other (voluntary work, takes care of household)	4 (5)
Income *n* (%)	
No income	4 (5)
EUR 1500 or less	30 (36)
EUR 1501 to 2500	23 (27)
EUR 2501 to 3500	21 (25)
EUR 3501 or more	4 (5)
Unknown	2 (2)
Income parents between 12 and 14 years *n* (%)	
Much lower than average	2 (2)
(Slightly) lower than average	20 (24)
Average	28 (33)
(Slightly) higher than average	25 (30)
Much higher than average	2 (2)
Unknown	7 (8)
BMI *mean (SD)*	26.6 (5.6)
Right handedness *n* (%)	70 (83)
Sensation threshold (mA) *mean* (SD)	2.1 (0.9)
Migration background^b^ *n* (%)	
Yes	27 (32)
No	57 (68)
TRAIT anxiety (scale 20–80) *mean* (SD)	35.2 (9.6)
Appreciation for music (7‐point Likert scale) *mean* (SD)	6.2 (1.1)
Appreciation for culture (7‐point Likert scale) *mean* (SD)	5.0 (1.5)
Playing of an instrument *n* (%)	
Yes	27 (32)
No	57 (68)

Abbreviations: mA, milliampere; SD, standard deviation; TRAIT, Trait Anxiety Inventory.

^a^ Level of education (currently pursuing or successfully completed) is based on the International Standard Classification of Education (UNESCO Institute for Statistics 2012). The Dutch synonyms are described more specifically in the protocol paper of this study.

^b^ Participant and/or at least one parent not born in the Netherlands.

### 3.1 MIA in the Context of Music Choice

**FIGURE 3:**
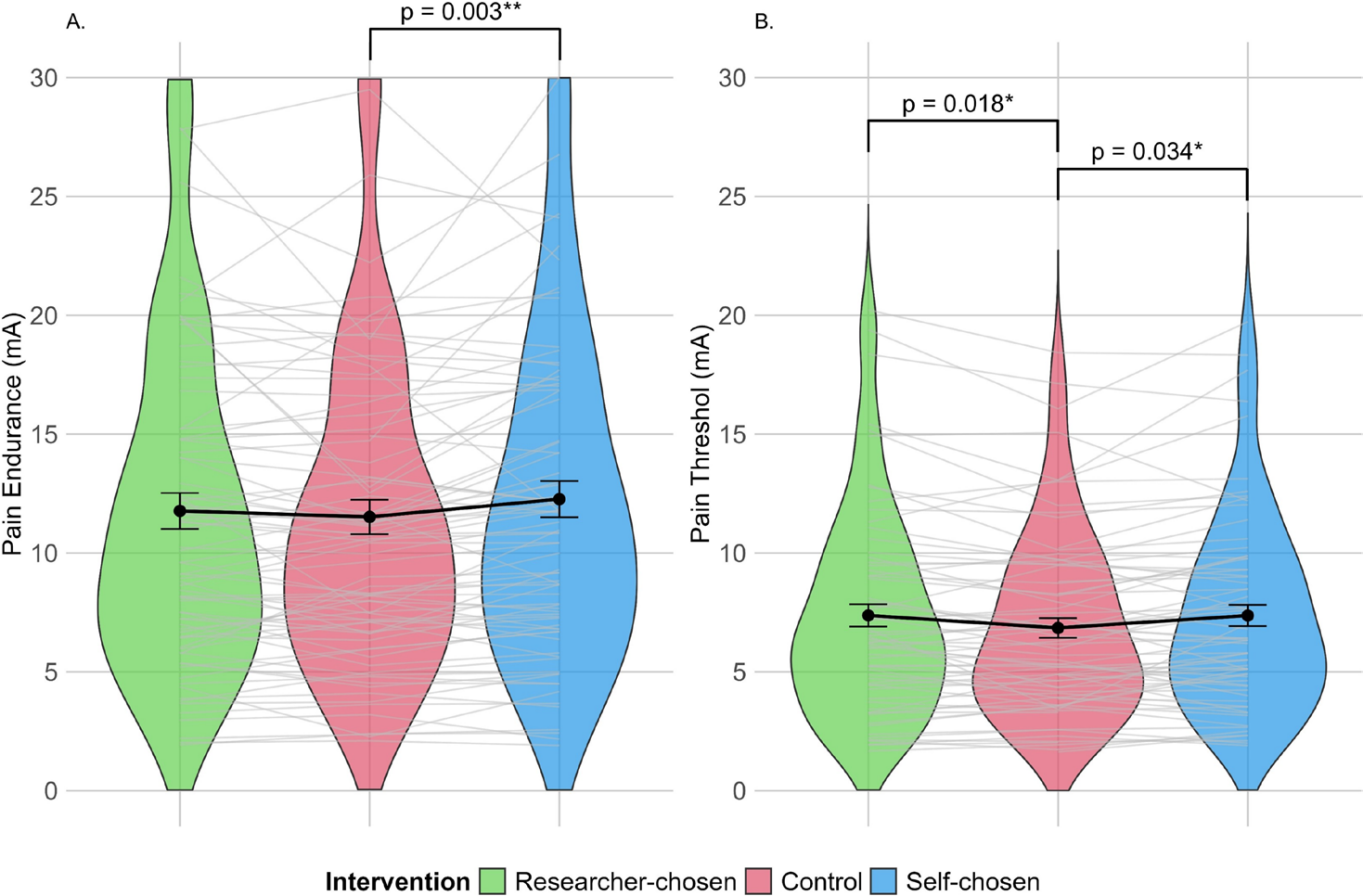
Pain endurance and pain threshold across interventions. The figures illustrate violin plots for pain endurance (A) and pain threshold (B). Per intervention group (control, researcher‐chosen music and self‐chosen music), the individual data points for each participant are connected by thin lines and the mean (±SD) values are displayed in bold lines. *p*‐values are based on linear mixed models, as summarised in Table 2, with the control set as the reference category. mA, milliampere, SD, standard deviation. *p < 0.05, **p < 0.01.

Pain measurement outcomes were analysed using LMMs, taking into account the sequence of the consecutive interventions in the crossover design (Table 2). The primary outcome, pain endurance, showed statistically significantly higher values for the self‐chosen music than for the control condition (*β* = 0.74 mA, 95% CI [0.26, 1.22], *p* = 0.003), whereas researcher‐chosen music was not significantly different (*β* = 0.27 mA, 95% CI [−0.21, 0.75], *p* = 0.274). The pain endurance and pain threshold for the three interventions are visualised in Figure 3. For the secondary pain outcomes, pain threshold was significantly greater for both researcher‐chosen music (*β* = 0.37 mA, 95% CI [0.07, 0.68], *p* = 0.018) and self‐chosen music (*β* = 0.32 mA, 95% CI [0.03, 0.62], *p* = 0.034) than for the control condition. Pain intensity scored lower with the self‐chosen music compared to the control condition (*β* = −0.43, 95% CI [−0.67, −0.20], *p* = < 0.001), which was not the case with the researcher‐chosen music (*β* = −0.03, 95% CI [−0.26, 0.20], *p* = 0.803). Similarly, pain unpleasantness was lower with the self‐chosen music (*β* = −0.38, 95% CI [−0.66, −0.11], *p* = 0.007) compared to the control condition, whereas researcher‐chosen music (*β* = −0.08, 95% CI [−0.35, 0.20], *p* = 0.595) did not yield this effect. Overall, the sequence of the consecutive interventions significantly impacted pain measures, with higher endurance/threshold values and lower pain intensity scores observed in the course of the measurements, independent of the type of intervention. Next, post hoc tests were conducted to compare the effects of the interventions with each other. These comparisons indicated no differences between self‐chosen and researcher‐chosen music in pain endurance, pain threshold or pain unpleasantness (Table S5). However, pain intensity was lower for self‐chosen music compared to both researcher‐chosen music (*β* = 0.40, 95% CI [0.13, 0.68], *p* = 0.002) and control (*β* = 0.43, 95% CI [0.16, 0.71], *p* < 0.001) in the post hoc test.

**TABLE 2:** Results of linear mixed models for pain measurements.

Outcome	Value	Intervention			Sequence effect
		Control	Researcher‐chosen music	Self‐chosen music	
Pain endurance (mA)	*β* (95% CI)	(ref.)	0.27 (−0.21; 0.75)	0.74 (0.26; 1.22)	0.25 (0.01; 0.49)
	*p*‐value	(ref.)	0.274	0.003**	0.044*
Pain threshold (mA)	*β* (95% CI)	(ref.)	0.37 (0.07; 0.68)	0.32 (0.03; 0.62)	0.22 (0.06; 0.37)
	*p*‐value	(ref.)	0.018*	0.034*	0.007**
Pain intensity (NRS)	*β* (95% CI)	(ref.)	−0.03 (−0.26; 0.20)	−0.43 (−0.67; −0.20)	0.16 (0.04; 0.28)
	*p*‐value	(ref.)	0.803	< 0.001***	0.008**
Pain unpleasantness (NRS)	*β* (95% CI)	(ref.)	−0.08 (−0.35; 0.20)	−0.38 (−0.66; −0.11)	0.10 (−0.04; 0.25)
	*p*‐value	(ref.)	0.595	0.007**	0.143

*Note:* Linear mixed models were conducted for the listed pain measurements under consideration of the intervention group (control, researcher‐chosen music, self‐chosen music) and the sequence of the interventions as part of the crossover design (sequence effect). The control intervention was set as the reference category. For pain threshold 80 unique participants were included, whereas for all other outcomes all 84 participants were included.

Abbreviations: CI, confidence interval; mA, milliampere; NRS, numeric rating scale.

* *p* < 0.05.

** *p* < 0.01.

*** *p* < 0.001.

### 3.2 MIA in the Context of Socio‐Cultural Background

**FIGURE 4:**
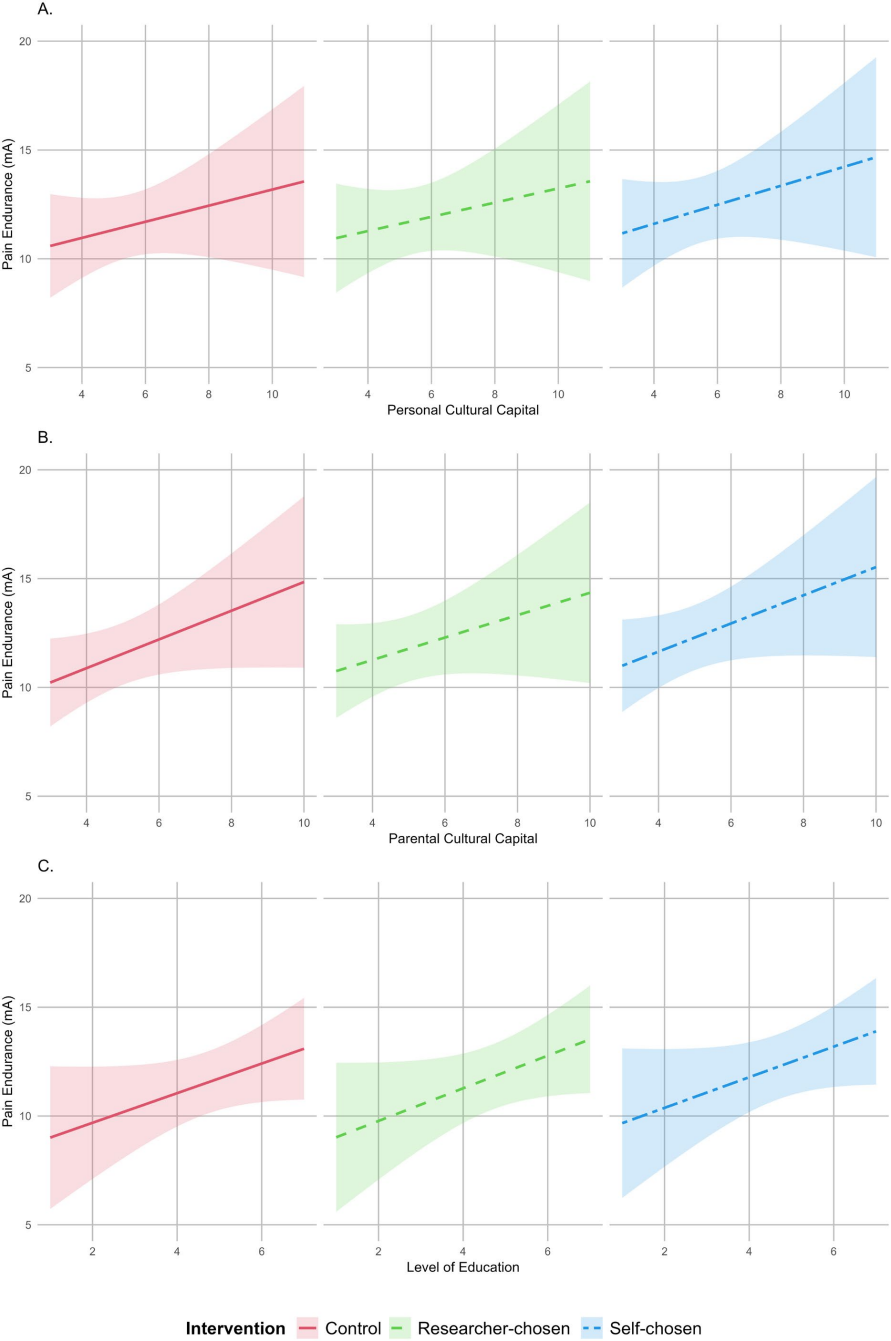
Influence of personal cultural capital, parental cultural capital and level of education on pain endurance per intervention. Influence of personal cultural capital (A), parental cultural capital (B) and level of education (C) on pain endurance for each intervention, modelled using simple linear regressions with 95% confidence intervals. Higher levels of education and cultural capital were associated with greater pain endurance. No statistically significant differences were found between intervention groups or socio‐cultural background parameters. mA, milliampere.

An exploratory approach was employed to investigate the influence of three socio‐cultural background parameters (personal cultural capital, parental cultural capital and level of education) on MIA across the different interventions. The distributions of these parameters are shown in Figure S1. Adding these parameters as interaction terms (independently and together) to the LMMs (paragraph 3.1) did not significantly impact the effect of the intervention on pain endurance or other pain measurements. However, this statistical approach could be limited by the number of terms and subjects, potentially leading to overfitting. Therefore, simple linear regressions were used to explore the influence of the socio‐cultural background parameters on pain endurance for the three interventions (Figure 4).

The distributions of cultural capital and level of education were balanced across randomisation groups (sequence of the auditory intervention), with no statistically significant differences between these groups. Overall, higher cultural capital and level of education showed a (statistically nonsignificant) trend toward increased pain endurance, irrespective of the type of intervention. The steepest slope was observed for researcher‐chosen music (0.75 mA, *p* = 0.080) in relation to level of education, compared with self‐chosen music (0.70 mA; *p* = 0.099) and control (0.68 mA, *p* = 0.094), with similar trends in personal and parental cultural capital. In summary, none of the differences between intervention groups and socio‐cultural background parameters were statistically significant. This suggests that the positive effect of self‐chosen music on pain endurance was present irrespective of personal and parental cultural capital and level of education.

### 3.3 Physiological and Psychological Outcomes

**FIGURE 5:**
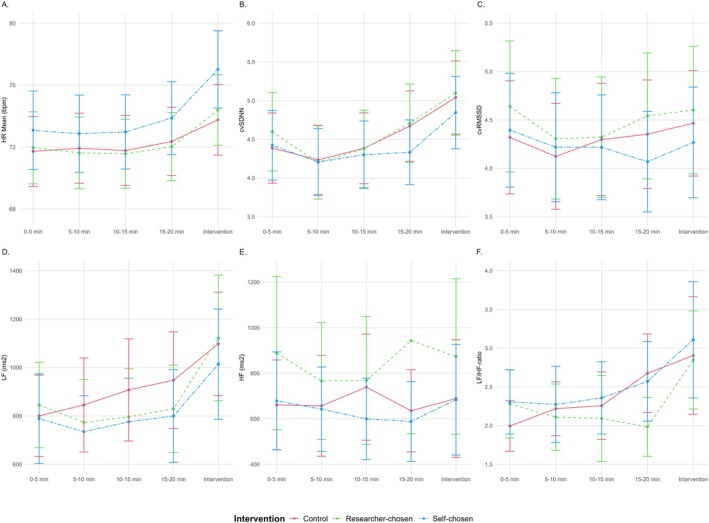
Standardised heart rate variability parameters per intervention. The figures illustrate the mean and 95% confidence intervals for the standardised HR mean (A), cvSDNN (B), cvRMSSD (C), LF (D), HF (E) and LF/HF ratio (F) over time, per intervention. Each measurement point corresponds to a 5‐min interval, with the pain stimulus occurring during the final interval. cv, coefficient of variation; HF, high frequency; HR, heart rate; LF, low frequency; RMSSD, root mean square of successive differences; SDNN, standard deviation of NN intervals.

Physiological and psychological outcomes were analysed using LMMs in line with the pain measurements analyses. The HRV parameters are visualised in 5‐min intervals for the three interventions in Figure 5. When examining these physiological parameters, the mean, minimum and maximum HR were significantly higher for the self‐chosen music as compared to the control condition (Table S6). Notably, cvRMSSD yielded higher values in the researcher‐chosen music (*β* = 0.2, 95% CI [0.0, 0.3], *p* = 0.009) compared to control, whereas cvSDNN did not show significant differences for both self‐ and researcher‐chosen music. Looking at the frequency domain, LF decreased with self‐chosen music (*β* = 60.6, 95% CI [28.9, 92.2], *p* < 0.001), whereas HF increased with researcher‐chosen music (*β* = 178.5, 95% CI [90.6, 266.4], *p* < 0.001) compared with the control condition. The LF/HF ratio did not exhibit significant differences. Overall, listening to self‐chosen music was associated with higher HR and lower LF values, whereas higher cvRMSSD and HF values indicated greater parasympathetic activity for the researcher‐chosen music.

With respect to the psychological outcomes (Table S7), state anxiety was significantly lower for self‐chosen music (*β* = −2.5, 95% CI [−3.8, −0.7], *p* < 0.001) and researcher‐chosen music (*β* = −1.5, 95% CI [−2.7, −0.7], *p* = 0.028) in comparison to the control condition. This effect was greater for self‐chosen music, suggesting that participants experienced the least anxiety while listening to their own choice of music. Additionally, participants rated their emotional state with the SAM (Figure S2). The self‐chosen music resulted in greater valence as compared to the control condition (*β* = 0.7, 95% CI [0.3, 1.0], *p* < 0.001). Dominance also yielded higher values for the self‐chosen music compared to the control group (*β* = 0.4, 95% CI [0.1, 0.2], *p* = 0.023), indicating that participants felt more in control. No significant differences between the music interventions and the control group were found for arousal, although a (statistically nonsignificant) trend suggested that participants felt calmer after listening to the researcher‐chosen music (*β* = 0.3, 95% CI [−0.0, 0.7], *p* = 0.081). Post hoc pairwise comparisons for the psychological measurements (Table S8) revealed no differences between self‐chosen and researcher‐chosen music for anxiety, valence, arousal or dominance. Moreover, using simple linear regressions to explore the influence of socio‐cultural background parameters on the psychological measures across the three interventions, no significant effects were found.

### 3.4 Characteristics of Interventions

First, the characteristics of the self‐chosen and researcher‐chosen music were analysed via the Spotify API (Table S9). Self‐chosen music showed especially higher values for energy, tempo (bpm) and danceability, compared to the researcher‐chosen music. Genres predominantly chosen by participants were pop (37%), rock (18%), rhythm‐and‐blues (14%), classical (6%) and hip‐hop (5%). The music genres varied across stratification groups: the more educated group selected classical music more often, whereas the less educated group chose (Dutch) pop more often. Second, the neutrally spoken podcast about flora and fauna was evaluated. Overall, 50% (*n* = 42) of participants liked the podcast, whereas 49% (*n* = 41) disliked it. This rating did not differ significantly between randomisation and stratification groups.

## 4 Discussion

The aim of this study was to investigate whether the effect of (self‐selected) music on pain endurance is consistent across different socio‐cultural backgrounds. Our findings indicate that self‐chosen music was effective in MIA (higher pain endurance and ‐threshold, lower perceived pain scores) irrespective of socio‐cultural background (cultural capital, level of education) and type of music. Self‐chosen music also resulted in increased sympathetic activity (higher HR), reduced anxiety and higher valence. Researcher‐chosen (classical) music was most effective in increasing parasympathetic activity (higher RMSSD and HF). Pairwise comparisons revealed that self‐chosen music led to lower pain intensity compared to ‘universal’ researcher‐chosen music, with no significant differences observed for any other pain measurement.

### 4.1 Effect of Music on Pain

Our study demonstrated that pain endurance was higher with self‐chosen music, which was not influenced by socio‐cultural background. This finding was in line with the other pain measurements (higher pain threshold, lower pain intensity, and lower pain unpleasantness). This confirms our first hypothesis, suggesting that the beneficial effect of self‐chosen music on pain remains consistent irrespective of socio‐cultural background. Previous research has indicated the crucial role of socio‐cultural background (level of education and cultural capital) in music taste formation and music perception (Bourdieu 1984; Hanser et al. 2016; Jæger and Møllegaard 2022; Lizardo and Skiles 2016; Sonnett 2020; Van Eijck 2001). However, up till now, while it has been well established that people with different backgrounds have different music tastes, it was not clear whether (tastes for) different kinds of music impact pain perception differently. Moreover, previous research on MIA did not include populations with diverse backgrounds representative of patients in healthcare (Lee 2016; Martin‐Saavedra et al. 2018). In our study, participants with diverse backgrounds indeed selected widely diverging types of music in terms of music genres and characteristics. Nonetheless, the effect of self‐chosen music on pain was consistent, indicating that the observed benefits of preferred music on MIA were driven by personal preference and/or free choice rather than the specific type of music. This finding supports and expands previous research that indicates that perceived control and personal preferences, irrespective of the type of music genre, are important contributors to MIA (Howlin and Rooney 2021a; Van der Valk Bouman et al. 2024). Interestingly, post hoc tests revealed a significant difference between self‐chosen and researcher‐chosen music only for pain intensity. It remains questionable to what extent this observation may have been influenced by factors such as the listening duration, the type of researcher‐chosen music, and the nature of the pain stimulus. For instance, a meta‐analysis on chronic pain reported greater analgesic effects of self‐chosen music, which was not the case for researcher‐chosen music (Garza‐Villarreal et al. 2017). However, direct comparisons remain limited in the literature, making it difficult to draw definitive conclusions (Colebaugh et al. 2023; Valevicius et al. 2023).

Based on our analysis, there is insufficient evidence to either support or reject the second hypothesis that researcher‐chosen (classical) music has a universal analgesic effect. Further research employing alternative statistical approaches (e.g., Bayesian inference) is needed to more thoroughly evaluate this hypothesis. While examining the interplay of MIA and social‐cultural background, it was notable that higher cultural capital and level of education showed a (statistically nonsignificant) trend toward greater pain endurance, with the highest slope observed for researcher‐chosen (classical) music. Arguably, this aligns with the concept of ‘highbrow’ culture in sociology, which asserts that individuals with higher cultural capital and level of education are more inclined to appreciate classical music (Lizardo and Skiles 2016). With some restraint due to the lack of statistical significance, the trend toward increased pain endurance among individuals with higher cultural capital and educational backgrounds may be explained by previous studies suggesting that highly educated individuals typically possess more effective coping mechanisms (Holahan and Moos 1987). However, the lack of statistical significance in this study limits the interpretation of cultural capital in relation to pain endurance, which warrants further research.

### 4.2 Mechanisms Underlying MIA

Interestingly, both self‐chosen and researcher‐chosen music resulted in a higher pain threshold compared to the podcast (control) condition. This suggests that music itself, regardless of its characteristics, genre and perceived control of music selection, has an analgesic effect that primarily influences the threshold at which a stimulus is perceived as painful. In line with previous research, participants reported a higher valence and lower anxiety with self‐chosen music (Hofbauer and Rodriguez 2023; Roy et al. 2008), which could have contributed to the effectiveness of self‐chosen music on pain endurance and perceived pain scores in our study. In contrast, researcher‐chosen music did not show significant differences in valence compared to either control or self‐chosen music. This may partially explain why researcher‐selected music showed no effect on pain endurance compared to both conditions. Other mechanisms underlying MIA could also explain the findings of this study (Akelma et al. 2024; Basinski et al. 2021; Lunde et al. 2019). Distraction is one mechanism frequently discussed in the literature, and the extent to which something is distracting might also be explained by factors such as familiarity and predictability (Arıcan and Soyman 2025; Howlin and Rooney 2020; Schäfer and Sedlmeier 2010). As the self‐chosen music was played in shuffle mode, this may have been the optimal combination of prediction and surprise to achieve the highest effectiveness (Daikoku et al. 2024; Gold et al. 2023; Ueno and Shimada 2024). Given that our control condition involved a podcast, it is unlikely that any auditory distraction alone accounts for the observed effects. Other potential mechanisms underlying the effectiveness of self‐chosen music in this study include neurotransmitter release and context factors such as expectations and a potential placebo effect (Cheung et al. 2024; Valevicius et al. 2023; Vuust et al. 2022).

### 4.3 Other Purposes and Types of Music

In terms of the HRV, greater parasympathetic activity (higher RMSSD and HF) was found with the researcher‐chosen music, whereas greater sympathetic activity (higher HR mean, HR min and HR max) was observed with the self‐chosen music as compared to the control condition. In line with this, participants tended to rate their arousal lowest (statistically nonsignificant) with the researcher‐chosen music. This more ‘relaxed’ state with the researcher‐chosen music, both self‐reported and measured using HRV, could be explained by the music characteristics. Compared to the researcher‐chosen music, self‐chosen music had overall higher energy, danceability and bpm, which has also been described in a previous study (Howlin and Rooney 2021b). Additionally, participants could have experienced the researcher‐chosen music as less stimulating and engaging, since this music was not necessarily in line with participants' individual preferences. Nevertheless, in clinical care, situations where it is beneficial for patients to achieve higher parasympathetic activity, such as before surgery, researcher‐chosen (relaxing classical) music could be an even more effective option than self‐chosen music.

### 4.4 Clinical Significance

The findings of this study have several clinical implications. First, our findings suggest that patients should be encouraged to select their own music for pain management, as self‐chosen music was effective in MIA across socio‐cultural backgrounds. Second, the observation that lower cultural capital and level of education showed a (statistically nonsignificant) trend toward lower pain endurance cautiously suggests that healthcare providers should be attentive to pain management in patients with these characteristics (Thurston et al. 2023). Third, the type of music, in terms of characteristics, genre, and by whom it is selected, could serve different functions. Whereas self‐chosen music was most effective for (acute) pain, researcher‐chosen (classical) music predicted higher parasympathetic activity. Hence, being aware of the intended function of the music intervention is crucial for patients and healthcare providers. Taken together, these findings demonstrate that personalised music selection should be encouraged in clinical care, with patients being entrusted with choosing the music that best suits their needs.

### 4.5 Strengths, Limitations and Directions for Future Research

A major strength of this study was the diverse study population in terms of socio‐cultural backgrounds. This is crucial since prior experimental research on MIA has typically been overrepresented by highly educated Western study cohorts (Becker et al. 2024; Lee 2016; Yi et al. 2025). However, recruiting enough participants, especially from lesser‐educated groups, was a significant challenge. As a result, this study's recruitment process was comprehensive and time‐consuming, as participants were generally uninterested in participating in scientific research. This reflects the effort required to ensure a truly diverse sample that is (more) representative of the patient population. Additionally, this study is the first to integrate sociological theories and methods into medical research on MIA, providing a unique perspective on this topic. Moreover, the use of a podcast as a control condition allowed us to control for the distraction component and draw more valid conclusions as compared to a control condition with silence. Employing other control conditions, such as natural sounds or visual media, could be interesting (Choi and Park 2022). Another strength was the use of a crossover design and LMMs, which accounted for individual differences, the sequence order of the conditions and improved the robustness of our findings. While accounting for the condition order as a covariate in the analyses, we found that the sequence effect itself was significant, highlighting the importance of considering such effects in both study design and interpretation. Additionally, only female participants were included, which made the pain measurements less biased by potential gender interactions, as all researchers in this study were also female.

However, this can also be seen as a limitation, as the results of this study are less transferable to men and people identifying as nonbinary, which should be investigated in future research. Another limitation lies in the fact that this study was the first to explore MIA in the context of socio‐cultural background and was not specifically powered to detect differences based on socio‐cultural background parameters due to the experimental design of this study. Although our exploratory analysis did not reveal significant differences in these parameters, further studies with larger sample sizes are needed to substantiate these findings. In this context, future research could also explore the potential of AI‐enabled, personalised music interventions in healthcare, taking socio‐cultural background parameters into account. Moreover, this study, along with many other studies on MIA, was conducted with healthy volunteers. Therefore, further research is needed in clinical settings with patients to validate these findings. As pain perception is a multidimensional and highly subjective experience, its measurement presents certain challenges. In this study, we used pain endurance as a more objective primary outcome, which may introduce bias, as participants can voluntarily stop the shock sequence. However, comparable methods often also face challenges in terms of bias or ethical concerns. Another consideration is that pain endurance is strongly linked to other measures such as pain intensity and unpleasantness. While these pain measures were generally consistent with one another, they are not independent, and this overlap should be considered when interpreting our findings. Finally, this study did not find evidence that self‐chosen music is superior to researcher‐chosen music, contrary to our expectations. It remains uncertain whether this outcome is due to the study design or specific music characteristics. Given the complex interplay between music features and individual preferences, it is difficult to disentangle their respective contributions. While our results suggest that self‐chosen music is effective, likely due to the alignment between personal choice and preference, the differing music characteristics between the self‐chosen and research‐chosen music may also have influenced the outcome. Future studies should aim to isolate these factors more precisely.

## 5 Conclusion

In conclusion, this study indicates that self‐chosen music is effective for MIA (higher pain endurance and ‐threshold, lower perceived pain scores), which was consistent irrespective of socio‐cultural background and type of music. Self‐chosen music also resulted in increased sympathetic activity (higher HR), reduced anxiety and greater valence. Researcher‐chosen (classical) music was most effective at increasing parasympathetic activity (higher RMSSD and HF). Hence, this study highlights that music is effective in MIA when it aligns with personal preferences, encouraging a personalised approach when using music for pain relief.

## Author Contributions

This study was designed by A.S.B., E.S.v.d.V.B., J.S., K.v.E., C.C.d.V. and M.K. The experiments were performed by A.S.B., E.S.v.d.V.B., Z.B. and C.S. The data were analysed by E.S.v.d.V.B. and A.S.B. and the results were critically examined by all authors. A.S.B. and E.S.v.d.V.B. had a primary role in preparing the manuscript, which was edited by J.S., K.v.E., Z.B., C.S., H.J., C.C.d.V. and M.K. All authors have approved the final version of the manuscript and agree to be accountable for all aspects of the work.

## Conflicts of Interest

The authors declare no conflicts of interest.

## Supporting information


**Data S1:** Supporting Information.

## Data Availability

All data and program codes are available upon reasonable request to the corresponding author.
