# Supplementary material for: Self‐Chosen Music as a Contributor to Music‐Induced Analgesia Across Diverse Socio‐Cultural Backgrounds: A Crossover Randomised Controlled Trial
Source: Eur J Pain. 2025 Aug 7;29(8):e70095. doi: 10.1002/ejp.70095 (PMC12330777; doi:10.1002/ejp.70095)
Supplement: Supplementary file 1 — Data S1: Supporting Information. [file EJP-29-0-s001.docx]

**SUPPLEMENTARY INFORMATION**

**TableS1 – Overview of the inclusion and exclusion criteria**

| **Inclusion criteria** | **Exclusion criteria** |
| --- | --- |
| Between 18 and 60 years of age | Significant hearing impairment |
| Female | Current complaints of tinnitus |
| Sufficient knowledge of Dutch language | Current treatment by a medical specialist or general practitioner |
| Provision of written informed consent | Current use of analgesic medication |
|  | Presence of acute or chronic pain |
|  | History of cardiac disease of arrhythmias |
|  | Electric implants (e.g. pacemakers) |
|  | Diagnosed psychiatric or neurological impairments |
|  | (Suspected) pregnancy |
|  | Significant hearing impairment |

**TableS2 – Research-chosen music playlist**

| **Song** | **Artist** | **Length** | **bpm** | **Popularity** | **Key** |
| --- | --- | --- | --- | --- | --- |
| Sonata No. 14 "Moonlight" in C-Sharp Minor", Op. 27 No. 2: I. Adagio sostenuto | Ludwig van Beethoven, Paul Lewis | 5:15 | 86 | 71 | C# minor |
| Cello Suite No. 1 in G Major, BWV 1007: I. Prélude | Johann Sebastian Bach, Yo-Yo Ma | 2:32 | 75 | 68 | G major |
| Clarinet Concerto in A major, K.622: II. Adagio | Wolfgang Amadeus Mozart – Martin Fröst, Deutsche Kammerphilharmonie Bremen | 6:40 | 67 | 49 | D major |
| Concerto in D Minor, BWV 974: II. Adagio | Johann Sebastian Bach, Víkingur Ólafsson | 4:10 | 64 | 47 | D minor |
| Nocturne No. 2 in E-Flat Major, Op. 9 No. 2 | Frédéric Chopin, Daniel Barenboim | 4:34 | 79 | 68 | Eb major |
| Minuet in A major, D. 334 | Franz Schubert, Arcadi Volodos | 3:13 | 92 | 64 | A major |

The researcher-chosen music was selected by our research group. Length, BPM, Popularity and Key were determined with the Spotify® Application Programming Interface.

Abbreviations: bpm= beats per minute.

**TableS3 – Overview of Spotify**® **audio features**

| **Audio feature** | **Description** |
| --- | --- |
| **Acousticness** | A confidence value indicating the likelihood of a track being acoustic (performed on nonamplified instruments), ranging between 0 and 1. |
| **Danceability** | A value indicating how suitable a track is for dancing, ranging between 0 and 1. Higher values indicate increased danceability. |
| **Energy** | A perceptual measure of intensity and activity in a track, ranging between 0 and 1. |
| **Instrumentalness** | A confidence value indicating the likelihood of a track containing no vocals, ranging between 0 and 1. Values above 0.5 likely indicate instrumental tracks. |
| **Liveness** | A confidence value indicating the likelihood of a track being performed live, such as detecting audience sounds in the recording. |
| **Loudness** | A value indicating the overall loudness of a track, ranging between −60 and 0 dB. Likely measured in LUFS or a similar perceptual scale. |
| **Speechiness** | A value indicating the presence of spoken words in a track. |
| **Tempo** | A value representing the speed or pace of the track, estimated by the average beat duration, expressed in beats per minute (BPM). |
| **Valence** | A value indicating how positively valenced a track is (from a Western perspective), ranging between 0 and 1. High values suggest a more positive, happy, and cheerful perception of the track. |

**TableS4 – Overview of heart rate variability parameters**

| **Parameter** | **Unit** | **Description** |
| --- | --- | --- |
| **Time domain** | | |
| HR mean | bpm | Mean heart rate |
| RMSSD | ms | Root Mean Square of Successive Differences |
| cvRMSSD | % | Coefficient of variance of RMSSD (division by mean beat-to-beat interval and multiplication by 100) |
| SDNN | ms | Standard deviation of NN intervals |
| cvSDNN | % | Coefficient of variance of SDNN (division by mean beat-to-beat interval and multiplication by 100) |
| **Frequency domain** | | |
| LF | ms^2^ | Absolute power of the low-frequency band (0.04–0.15 Hz) |
| HF | ms^2^ | Absolute power of the high-frequency band (0.15–0.4 Hz) |
| LF/HF-ratio | % | Ratio of LF-to-HF power |

The heart rate variability parameters overview is based on Shaffer et al. 2017. All parameters were calculated using Kubios HRV Scientific (version 4.1.2). The frequency domain parameters were calculated via fast Fourier transformation.

Abbreviations: HR=heart rate; cv=coefficient of variation; SDNN=standard deviation of NN intervals; RMSSD=root mean square of successive differences; LF=low frequency; HF=high frequency; ms=milliseconds.

**TableS5 – Pairwise comparisons of pain measurements**

| **Measure** | **Comparison** | | **β (95% CI)** | **p-value** |
| --- | --- | --- | --- | --- |
| **Pain endurance (mA)** | Control | Researcher-chosen | -0.27 (-0.85; 0.31) | 0.513 |
|  | Control | Self-chosen | -0.74 (-1.32; -0.16) | 0.009** |
|  | Researcher-chosen | Self-chosen | -0.47 (-1.95; 0.11) | 0.154 |
| **Pain threshold (mA)** | Control | Researcher-chosen | -0.37 (-0.75; -0.00) | 0.048* |
|  | Control | Self-chosen | -0.32 (0.09; -0.68) | 0.089 |
|  | Researcher-chosen | Self-chosen | 0.05 (-0.32; 0.42) | 0.947 |
| **Pain intensity (NRS 0-10)** | Control | Researcher-chosen | 0.03 (-0.25; 0.31) | 0.966 |
|  | Control | Self-chosen | 0.43 (0.16; 0.71) | <0.001*** |
|  | Researcher-chosen | Self-chosen | 0.40 (0.13; 0.68) | 0.002** |
| **Pain unpleasantness (NRS 0-10)** | Control | Researcher-chosen | 0.08 (-0.26; 0.41) | 0.856 |
|  | Control | Self-chosen | 0.38 (0.05; 0.72) | 0.019* |
|  | Researcher-chosen | Self-chosen | 0.31 (-0.02; 0.64) | 0.075 |

Post hoc tests were conducted to analyze the differences between the three interventions (researcher-chosen music, self-chosen music, control) following the primary analyses using LMMs under consideration of the sequence.

Abbreviations: mA=milliampere; NRS=numeric rating scale. *p<0.05, **p<0.01, ***p<0.001.

**TableS6 – Results of linear mixed models for physiological outcomes**

| **Outcome** | **Value** | **Intervention** | | | **Sequence effect** | |
| --- | --- | --- | --- | --- | --- | --- |
|  |  | **Control** | **Researcher-chosen music** | **Self-chosen music** | **HRV intervals** | **Interventions** |
| **HR mean (bpm)** | β (95% CI) | (ref.) | -0.2 (-0.7; 0.2) | 1.8 (1.4; 2.2) | 0.7 (0.4; 0.9) | -2.4 (-2.7; -2.2) |
|  | p-value | (ref.) | 0.327 | <0.001*** | <0.001*** | <0.001*** |
| **HR min (bpm)** | β (95% CI) | (ref.) | 0.2 (-0.2; 0.7) | 1.7 (1.3; 2.1) | 0.28 (0.1; 0.5) | -2.1 (-2.3; -1.9) |
|  | p-value | (ref.) | 0.282 | <0.001*** | 0.002** | <0.001*** |
| **HR max (bpm)** | β (95% CI) | (ref.) | -0.2 (-1.0; 0.6) | 1.9 (1.1; 2.7) | 0.9 (0.5; 1.3) | -2.5 (-2.9; -2.1) |
|  | p-value | (ref.) | 0.585 | <0.001*** | <0.001*** | <0.001*** |
| **cvSDNN** | β (95% CI) | (ref.) | 0.1 (-0.1; 0.2) | -0.1 (-0.2; 0.0) | 0.1 (0.1; 0.2) | 0.1 (0.0; 0.1) |
|  | p-value | (ref.) | 0.320 | 0.096 | <0.001*** | 0.020** |
| **cvRMSSD** | β (95% CI) | (ref.) | 0.2 (0.0; 0.3) | -0.1 (-0.2; 0.1) | 0.0 (-0.1; 0.1) | 0.2 (0.1; 0.3) |
|  | p-value | (ref.) | 0.009** | 0.370 | 0.958 | <0.001*** |
| **LF (ms^2^)** | β (95% CI) | (ref.) | -29.8 (-108.0; 48.4) | -88.5 (-166.0; -11.1) | 60.6 (28.9; 92.2) | 8.0 (-31.3; 47.2) |
|  | p-value | (ref.) | 0.455 | 0.025* | <0.001*** | 0.689 |
| **HF (ms^2^)** | β (95% CI) | (ref.) | 178.5 (90.6; 266.4) | -32.2 (-119.3; 54.9) | 1.8 (-41.3; 44.9) | 90.8 (46.7; 134.9) |
|  | p-value | (ref.) | <0.001*** | 0.468 | 0.934 | <0.001*** |
| **LF/HF-ratio (%)** | β (95% CI) | (ref.) | -0.1 (-0.4; 0.1) | 0.1 (-0.1; 0.4) | 0.2 (0.1; 0.3) | -0.1 (-0.2; 0.1) |
|  | p-value | (ref.) | 0.339 | 0.357 | <0.001*** | 0.267 |

Linear mixed models were conducted for the listed physiological outcomes under consideration of the intervention group (researcher-chosen music, self-chosen music, control), the sequence of the HRV measurements (5-minute intervals) and the sequence of the three consecutive interventions. Based on information criteria and the log-likelihood test, a random slope for the sequence of the auditory interventions was included in the model. The control condition was set as the reference category.

Abbreviations: HRV=heart rate variability; HR=heart rate; cv=coefficient of variation; SDNN=standard deviation of NN intervals; RMSSD=root mean square of successive differences; LF=low frequency; HF=high frequency; ms=milliseconds. *p<0.05, **p<0.01, ***p<0.001.

**TableS7 – Results of linear mixed models for psychological outcomes**

| **Outcome** | **Value** | **Intervention** | | | **Sequence effect** |
| --- | --- | --- | --- | --- | --- |
|  |  | **Control** | **Researcher-chosen music** | **Self-chosen music** |  |
| **Anxiety (STAI-6)** | β (95% CI) | (ref.) | -1.5 (-2.7; 0.7) | -2.5 (-3.8; 0.7) | -0.6 (-1.3; 0.0) |
|  | p-value | (ref.) | 0.028* | <0.001*** | 0.066 |
| **Valence (SAM)** | β (95% CI) | (ref.) | 0.3 (-0.1; 0.6) | 0.7 (0.3; 1.0) | 0.2 (-0.0; 0.3) |
|  | p-value | (ref.) | 0.173 | <0.001*** | 0.102 |
| **Arousal (SAM)** | β (95% CI) | (ref.) | 0.3 (-0.0; 0.2) | -0.1 (-0.4; 0.2) | 0.1 (-0.1; 0.1) |
|  | p-value | (ref.) | 0.081 | 0.755 | 0.462 |
| **Dominance (SAM)** | β (95% CI) | (ref.) | 0.1 (-0.2; 0.2) | 0.4 (0.1; 0.2) | -0.0 (-0.2; 0.1) |
|  | p-value | (ref.) | 0.421 | 0.023* | 0.623 |

Linear mixed models were conducted for the listed psychological outcomes under consideration of the intervention group (researcher-chosen music, self-chosen music, control) and the sequence of the consecutive interventions. The control condition was set as the reference category.

Abbreviations: STAI=State-Trait Anxiety Inventory; CI=confidence interval; SAM=Self-Assessment Manikin. *p<0.05, **p<0.01, ***p<0.001.

**TableS8 – Pairwise comparisons of psychological outcomes**

| **Measure** | **Comparison** | | **β (95% CI)** | **p-value** |
| --- | --- | --- | --- | --- |
| **Anxiety (STAI-6)** | Control | Researcher-chosen | 1.45 (-0.10; 2.99) | 0.072 |
|  | Control | Self-chosen | 2.52 (0.96; 4.08) | <0.001*** |
|  | Researcher-chosen | Self-chosen | 1.08 (-0.48; 2.64) | 0.235 |
| **Valence (SAM)** | Control | Researcher-chosen | -0.26 (-0.71; 0.19) | 0.360 |
|  | Control | Self-chosen | -0.67 (-1.11; -0.22) | 0.002** |
|  | Researcher-chosen | Self-chosen | -0.41 (-0.85; -0.42) | 0.085 |
| **Arousal (SAM)** | Control | Researcher-chosen | 0.33 (-0.12; 0.79) | 0.188 |
|  | Control | Self-chosen | -0.06 (-0.51; 0.39) | 0.948 |
|  | Researcher-chosen | Self-chosen | -0.39 (-0.84; 0.06) | 0.098 |
| **Dominance (SAM)** | Control | Researcher-chosen | -0.13 (-0.50; 0.25) | 0.670 |
|  | Control | Self-chosen | -0.36 (-0.73; 0.01) | 0.059 |
|  | Researcher-chosen | Self-chosen | -0.23 (-0.61; 0.14) | 0.301 |

Post hoc tests were conducted to analyze the differences between the three interventions (researcher-chosen music, self-chosen music, control) following the primary analyses using LMMs under consideration of the sequence.

Abbreviations: mA=milliampere; STAI=State-Trait Anxiety Inventory; SAM=Self-Assessment Manikin. *p<0.05, **p<0.01, ***p<0.001.

**TableS9 – Characteristics of researcher- and self-chosen music**

| **Music feature (mean ± SD)** | **Researcher-chosen music** | **Self-chosen music** |
| --- | --- | --- |
| Acousticness | 0.97 ± 0.04 | 0.35 ± 0.33 |
| Danceability | 0.30 ± 0.12 | 0.56 ± 0.17 |
| Energy | 0.04 ± 0.06 | 0.57 ± 0.25 |
| Instrumentalnes | 0.84 ± 0.18 | 0.09 ± 0.24 |
| Liveness | 0.12 ± 0.05 | 0.17 ± 0.16 |
| Loudness | -30.29 ± 7.98 | -8.94 ± 5.00 |
| Speechiness | 0.05 ± 0.01 | 0.07 ± 0.07 |
| Tempo (bpm) | 99.24 ± 42.45 | 119.32 ± 28.49 |
| Valence | 0.14 ± 0.10 | 0.48 ± 0.27 |

Music features were analyzed via the Spotify® API. The mean and standard deviation were calculated using weights to account for differences in the number of tracks chosen by participants. A description of all Spotify® audio features is provided in **Supplementary Table 3**.

Abbreviations: bpm=beats per minute, SD=standard deviation.

FigureS1 – Histograms of personal and parental cultural capital and level of education


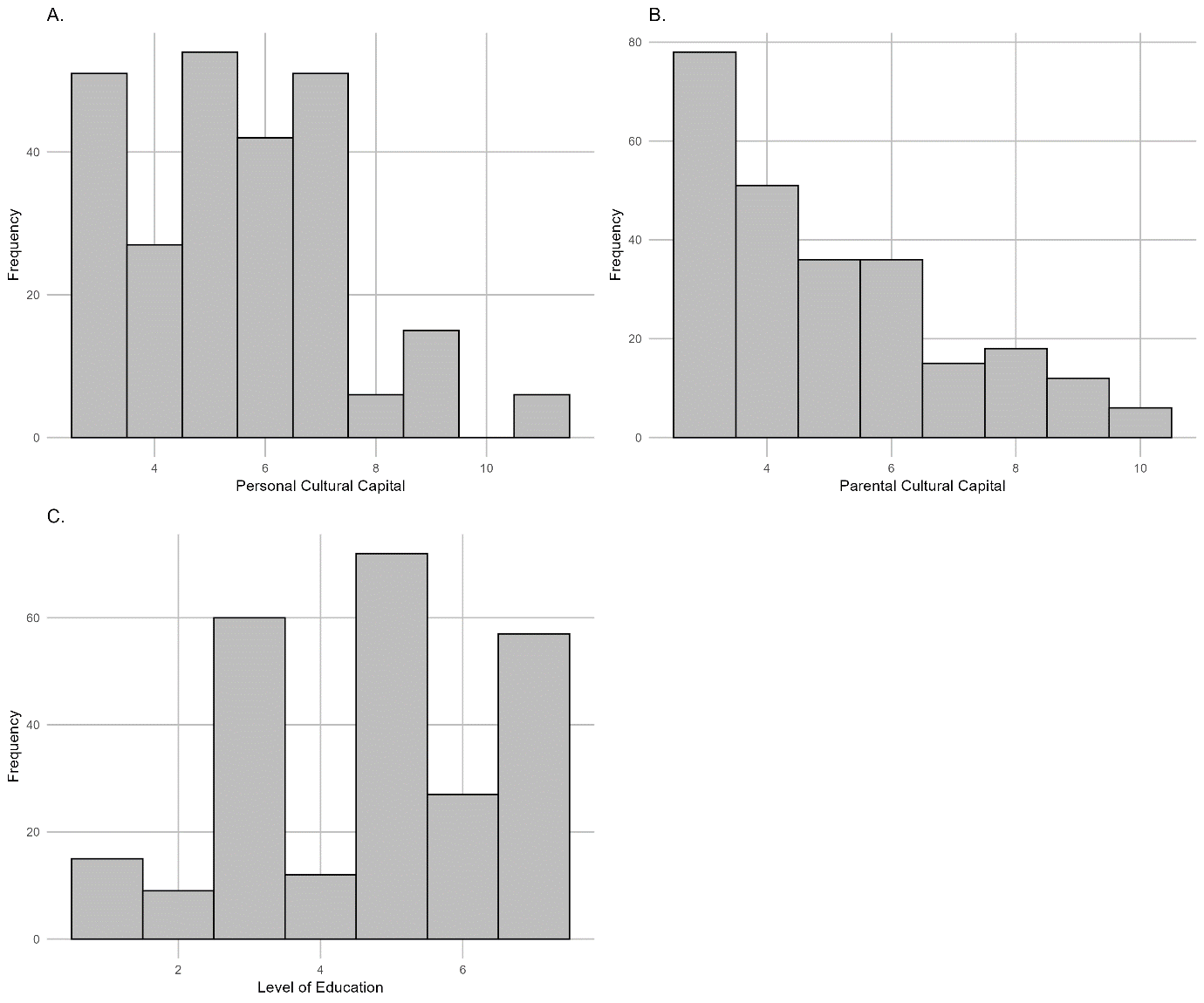


Histograms of personal cultural capital (**A**), parental cultural capital (**B**) and level of education (**C**). The histograms visualize the frequency for the total study population (n=84). Personal and parental cultural capital were based on 3 items (7-point Likert scale), with values potentially ranging from 3-21 in total. Level of education was measured on a scale from 1-7. The distributions were not significantly different between stratification and randomization groups.

FigureS2 – Russell’s Scale of Valence and Arousal mean values per intervention


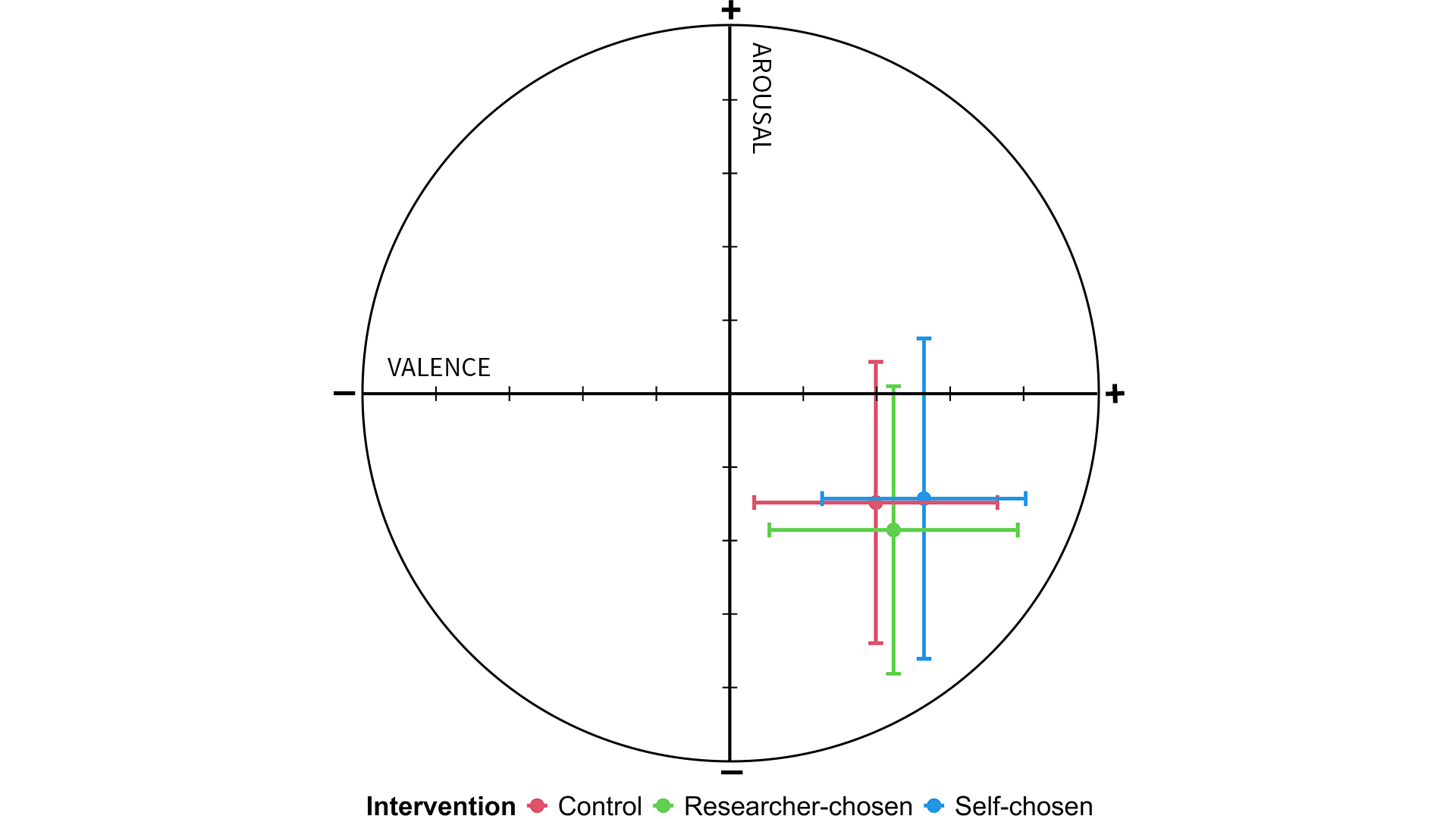


Valence and arousal, measured directly after the pain stimuli for the three interventions. Both scales range from 1 to 9, where 1 implies very low and 9 implies very high valence/arousal.
